# Recently Discovered Adipokines and Cardio-Metabolic Comorbidities in Childhood Obesity

**DOI:** 10.3390/ijms151119760

**Published:** 2014-10-29

**Authors:** Gloria Maria Barraco, Rosa Luciano, Michela Semeraro, Pedro L. Prieto-Hontoria, Melania Manco

**Affiliations:** 1 Research Unit for Multi-Factorial Diseases, Obesity and Diabetes, Scientific Directorate, Bambino Gesù Children Hospital, Rome 00146, Italy; E-Mails: gloria.barraco@yahoo.it (G.M.B.); semeraro.michela@gmail.com (M.S.); 2 Department of Laboratory Medicine, Bambino Gesù Children Hospital, Rome 00146, Italy; E-Mail: rosalucia81@tin.it; 3 Faculty of Health and Physical Activity Science, University (SEK), Santiago de Chile, Chile; E-Mail: pedro.prieto@usek.cl

**Keywords:** adipose tissue, adipokines, cardiovascular disease, childhood obesity, inflammation, metabolic syndrome

## Abstract

White adipose tissue (WAT) asset, in terms of cell number, fat storage capacity and endocrine function, is largely determined in early stages of life and is pivotal for shaping the WAT pro-inflammatory behavior. WAT derived adipokines have been shown to play a main role in several cardio-metabolic abnormalities of obesity. This review focuses on the most recently identified adipokines, namely adipocyte-fatty acid-binding protein, chemerin, fibroblast growth factor-21, lipocalin-2, omentin-1 and vaspin; their role in the pathogenesis of obesity and associated cardio-metabolic abnormalities; and on their adaptive response to body weight change. Evidence consistently suggests a pathogenic role for A-FABP, chemerin and FGF-21. Nevertheless, large population studies are needed to verify whether they can be useful to predict the risk of cardio-metabolic abnormalities in adulthood and/or monitor the clinical response to therapeutic interventions.

## 1. Introduction

Given the obesity epidemic in youth, the deeper comprehension of molecular mechanisms under its early onset and progression is foreseen. The severity of obesity seems to be one of the factors explaining the increased prevalence of cardio-metabolic abnormalities even if a valid definition with standardized cut-off values of the metabolic syndrome and the cardio-vascular risk assessment is not yet available in children. Adaptive changes in anatomy and physiology of the white adipose tissue (WAT) plays a major role in the natural history of childhood obesity and, particularly, of associated cardio-metabolic abnormalities [1]. WAT asset, in terms of cell number, fat storage capacity and endocrine function, is largely and firmly established in early stages of life, contributing to shape WAT pro-inflammatory phenotype [2]. WAT derived molecules, collectively called adipokines, have been shown to act as main players in several sub-clinical (*i.e.*, low-grade inflammation, insulin resistance—IR) and clinical cardio-metabolic abnormalities (*i.e.*, elevated blood pressure—BP, dyslipidemia, type 2 diabetes mellitus—T2DM and atherosclerosis) of obesity that can be tracked from a young age to adulthood, leading to overt cardiovascular disease even in youth [3]. Although the list of adipose-tissue derived pro-inflammatory factors has been lengthening in the last few years, evidence supporting their use as part of clinical practice in childhood obesity is not convincing. Adipokines could serve as biomarkers when they are demonstrated to function as biological indicators of mechanisms involved in a specific pathological development condition and progression with or without a casual relationship [3]. They serve as risk factors when they identify and predict a defined outcome of the disease and they are directly and casually connected with it [3]. In the present review, we will report on findings from studies in overweight and obese children (Table 1) dealing with the most recently discovered adipokines. We will focus on their role in the pathogenesis of obesity, associated morbidities and adaptive response to body weight change. Conversely, we will not discuss those adipokines such as leptin, adiponectin, retinol binding protein 4, visfatin and resistin that were identified a long time ago and excellently reviewed elsewhere [3,4]. 

Publications and cross references were extracted from the PubMed database (service of the United States National Library of Medicine that includes citations from MEDLINE and other life science journals for biomedical articles). Human studies on subjects younger than 18 years old concerning adipokines in childhood obesity were searched systematically (latest update 6 July 2014). Key search words were adipokines or their acronyms (adipocytes-fatty acid-binding protein (A-FABP or FABP4); chemerin (RARRES2 or TIG2); fibroblast growth factor-21 (FGF-21); lipocalin-2; omentin-1; vaspin); insulin resistance; inflammation; obesity; metabolic syndrome, cardiovascular disease; type II diabetes mellitus; non alcoholic fatty liver disease; articles in English and Spanish have been included in the analysis.

**Table 1:** Summary of studies about the most recently discovered adipokines.

Adipokine	Author [Ref.]	Population and Design	Main Findings
**A-FABP**	Corripio *et al.* [5]	At baseline: 73 obese *vs.* 47 normal-weight children aged 6–10 years; At 2 years: 31 obese patients lost weight; prospective cohort interventional study (2 years).	At baseline: 35.2 ± 14.6 *vs.* 10.4 ± 4.54 ng/mL (*p* < 0.001); At 2 years: in weigh losers from 37.3 ± 18.4 at baseline to 24.4 ± 13.9 ng/mL at 2 years (*p* < 0.001)
	Choi *et al.* [6]	At baseline: 48 overweight *vs.* 111 normal-weight children aged 9 years. At 3 years: 55 overweight *vs.* 104 normal-weight children of whom 10 boys developed MetS; prospective cohort observational study (3 years).	At baseline: 23.6 ± 8.2 *vs.* 12.8 ± 5.1 μg/L (*p* < 0.05); At 3 years: 25.9 ± 10.5 in those who developed MetS *vs.* 15.6 ± 7.4 μg/L (*p* < 0.001)
	Yun *et al.* [7]	161 children aged 9 years: 80 boys: 12 overweight *vs.* 25 at overweight *vs.* 43 normal weight and 81 girls: 13 overweight *vs.* 23 overweight *vs.* 43 normal-weight; cross-sectional observational study.	In boys: 22.3 ± 8.7 *vs.* 14.4 ± 5.2 *vs.* 8.5 ± 3.7 ng/mL; In girls: 24.4 ± 8.7 *vs.* 16.0 ± 7.9 *vs.* 7.8 ± 4.3 ng/mL (*p* < 0.01 for both)
	Krzystek-Korpacka *et al.* [8]	At baseline: 87 obese *vs.* 27 overweight *vs.* 31 normal-weight children aged 10–17 years; At 1 year: 84 patients under weight loss program and/or metformin treatment; prospective cohort interventional study (1 year).	Mean (95 CI%): 48.2 (44.5–51.9) at baseline *vs.* 33.2 (30.1–36.3) μg/L at 1 year (*p* < 0.001)
	Reinehr *et al.* [9]	At baseline: 30 obese *vs.* 10 normal-weight children aged 8–15 years; At 1 year: 10 lost weight; prospective cohort interventional study (1 year).	At baseline: increased A-FABP levels in obese children (data not reported, *p* = 0.009). Median and (IQR) at 1 year: in weigh losers from 41 (31–49) at to 29 (20–37) μg/L at 1 year (*p* < 0.01)
	Schipper *et al.* [10]	60 obese *vs.* 30 normal-weight children aged 6–16 years; cross-sectional observational study.	Median (IQR): 24.0 (21.5–27.0) *vs.* 23.6 (20.5–27.9) ng/mL (*p* = NS)
	Reyman *et al.* [11]	36 25(OH)D-deficient *vs.* 28 (in)sufficient obese children aged 6–16 years and 28 obese *vs.* 27 normal-weight 25(OH)D (in)sufficient children aged 6–16 years; cross-sectional observational study.	Median (IQR): 23.0 (20.9–26.4) *vs.* 25.7 (22.6–27.3) *vs.* 22.8 (20.4–27.6) ng/mL (*p* = NS)
**Chemerin**	Schipper *et al.* [10]	60 obese *vs.* 30 normal-weight children aged 6–16 years; cross-sectional observational study.	Median (IRQ): 3.0 ± 0.5 *vs.* 2.8 ± 0.4 µg/mL (*p* < 0.05)
	Reyman *et al.* [11]	36 25(OH)D-deficient *vs.* 28 (in)sufficient obese children aged 6–16 years and 28 obese *vs.* 27 normal-weight 25(OH)D (in)sufficient children aged 6–16 years; cross-sectional observational study.	Median (IQR): 3.13 (2.74–3.47) *vs.* 2.87 µg/mL (2.50–3.11) (*p* < 0.05);2.87 (2.50–3.11) *vs.* 2.80 µg/mL (2.48–3.00) (*p* = NS)
	Landgraf *et al.* [12]	105 obese *vs. *69 normal-weight children aged 7–18 years; cross-sectional observational study.	117.82 ± 26.4 *vs. *89.8 ± 16.1 ng/mL (*p* < 0.001)
**FGF-21**	Reinehr *et al.* [13]	At baseline: 60 obese *vs.* 40 normal-weight children aged 12–15 years; At 1 year: obese children with decreased SDS-BMI; prospective cohort interventional study (1 year).	Median (IQR): 195 (114–347) *vs.* 56 (33–122) pg/mL (*p* < 0.001); In weight loser from 206 (98–406) at baseline to 1 year 139 pg/mL (66–307) at 1 year (*p* = 0.038)		
	Giannini *et al.* [14]	79 obese with high hepatic fat fraction (HFF% ≥ 5.5%) *vs. *107 obese with HFF% < 5.5% *vs.* 31 normal-weight with HFF% < 5.5% adolescents aged 14–17 years; cross-sectional observational study.	277 ± 21 *vs.* 135 ± 8 *vs.* 99 ± 12 pg/mL (*p* < 0.001)		
**Lipocalin-2**	Corripio *et al.* [5]	At baseline: 73 obese *vs.* 47 normal-weight children aged 6–10 years; At 2 years: 31 weight losers; prospective cohort interventional study (2 years).	At baseline: 50.7 ± 18.4 *vs.* 28.0 ± 7.75 ng/mL (*p* < 0.001)*.* At 2 years: in weight losers from 48.4 ± 18.4 to 50.7 ± 23.4 ng/mL (*p* = 0.875)		
	Akelma *et al.* [15]	33 obese *vs.* 34 normal-weight children aged 9–14 years; cross-sectional observational study.	103.72 ± 41.26 *vs. *94.03 ± 49.61 ng/mL (*p* = NS)		
	Kanaka-Gantenbein *et al.* [16]	20 morbidly obese *vs.* 20 obese *vs.* 20 overweight *vs.* 20 normal-weight girls aged 9–16 years; cross-sectional observational study.	Mean (SD): 16.3 (3.7) *vs.* 12.2 (1.7) *vs.* 17.5 (3.1) *vs.* 23.3 (4.6) μg/L; (*p* < 0.05 between obese and normal-weight)		
**Omentin-1**	Schipper *et al.* [10]	60 obese *vs. *30 normal-weight children aged 6–16 years; cross-sectional observational study.	Median (IQR): 4.0 (3.5–4.5) *vs. *3.8 (3.3–4.4) pg/mL (*p* = 0.032)		
	Reyman *et al.* [11]	36 25(OH)D-deficient *vs.* 28 (in)sufficient obese children aged 6–16 years and 28 obese *vs.* 27 normal-weight 25(OH)D (in)sufficient children aged 6–16 years; cross-sectional observational study.	Median (IQR): 4.06 (3.43–4.55) *vs.* 3.81 (3.32–4.53) *vs.* 3.79 (3.33–4.40) pg/mL		
	Catli *et al.* [17]	49 obese *vs. *30 normal-weight children aged 6–14 years; cross-sectional observational study.	24.3 ± 9.8 *vs. *29.0 ± 6.7 ng/mL (*p* = 022)		
**Vaspin**	Ko *et al.* [18]	82 overweight *vs.* 86 normal-weight boys aged 9 years and 86 overweight *vs.* 90 normal-weight girls aged 9 years; cross-sectional observational study.	0.33 ± 0.59 *vs.* 0.18 ± 0.26 ng/mL (*p* < 0.05); 0.28 ± 0.52 *vs.* 0.17 ± 0.13 ng/mL (*p* < 0.05)		
	Suleymanoglu *et al.* [19]	33 obese *vs. *36 normal-weight children aged 11–16 years; cross-sectional observational study.	0.64 ± 0.3 *vs.* 0.42 ± 0.24 μg/L (*p* = 0.002)		
	Korner *et al.* [20]	67 obese *vs.* 65 normal-weight aged 7–19 years; cross-sectional observational study.	0.55 ± 0.06 *vs.* 0.92 ± 0.14 ng /mL (*p* = 0.013)		
	Martos Moreno *et al.* [21]	100 obese *vs.* 42 normal-weight children aged 5–13 years; prospective cohort interventional study (duration not reported).	At baseline 0.34 ± 0.34 *vs.* 0.34 ± 0.33 ng/mL (*p* = NS); no significant change after either 1 or 2 SDS BMI reduction		

## 2. Adipose Tissue and Inflammation in Childhood Obesity

**Figure 1:**
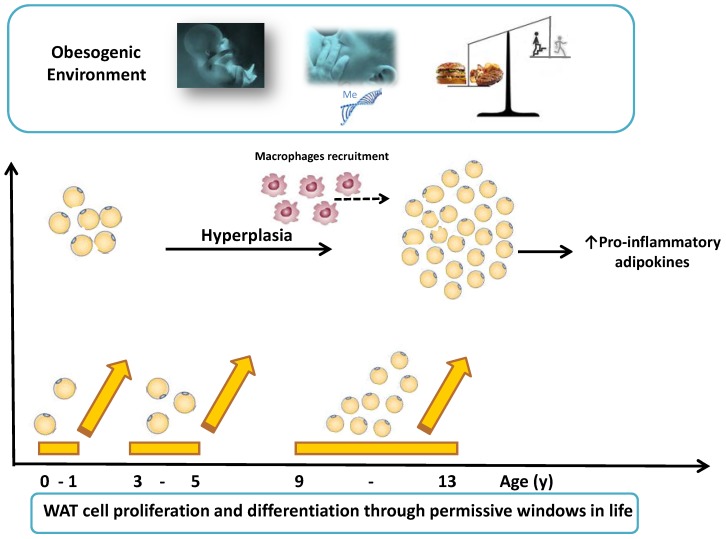
Permissive windows early in life. “Obesogenic” cues can enhance WAT hyperplasia. They encompass, for instance, hormonal stimuli *(i.e.*, endocrine disruptors) and nutrient availability during the intrauterine life, maternal epigenome, gut microbiota soon after the birth, early feeding and weaning, physical activity and sedentary behaviors.

WAT is naturally devoted to storing fat and energy but it also acts as an endocrine organ. WAT enlargement is due to the combination of an increase in number (hyperplasia) and volume (hypertrophy) of adipocytes. Owing to the mass effect, WAT releases a large amount of inflammatory molecules, either hormones or growth factors and cytokines, collectively called “adipokines” [2]. However, not only adipocytes but also stromo-vascular and immune cells (*i.e.*, macrophages and T-cells) embedded within WAT contribute releasing inflammatory molecules [22]. Within the fat-engorged adipose tissue, adipocyte-derived molecules can induce the recruitment of immune cells. These promote the recruitment and differentiation from pre-adipocytes to mature cells by releasing pro-inflammatory mediators in a vicious cycle [22]. Childhood obesity has been classically regarded so far as due prevalently to the hyperplasia of WAT, which is a highly “plastic” organ at this age [2]. The plasticity of the adipocytes is well defined in their capability of adaptation, in terms of number and volume, to the fat stores. There are “permissive windows” during life, when cells, tissues and organs are still responsive to external cues. The first year of life [23], the age between 3 to 5 (known as the “adiposity rebound period”) [24], and 9 to 13 (the “pubertal time”) [25] have been proposed as especially sensitive periods for adipocytes proliferation and differentiation, being WAT characterized by fast rate of growth and development (Figure 1) [2]. Adipose tissue is either white or brown. Brown adipose tissue (BAT) is involved in the thermogenic processes due to its higher expression of uncoupling protein-1 [26]. BAT was classically known to be active just in neonates, with its activity declining shortly thereafter. Recent findings established that BAT activity increases from childhood into adolescence, peaking by 13 years [27] and that functional deposits of BAT have been found in adult humans, thus overturning the classical view [3]. Previous studies have revealed BAT endocrine function, but further studies are needed to understand the mechanisms under this function and its ability to modify WAT and its derived factors and/or its link with obesity complications [28]. Starting from infancy, excess fat overflows, at first from subcutaneous (SAT) to visceral adipose tissue (VAT) [29], subsequently, the hypertrophic response of the VAT becomes prevalent and the ratio between SAT and VAT starts decreasing. Indeed, VAT has not been found to be associated with cardio-metabolic risk in obese prepubertal children [30]; while a reduced ratio has been related to more severe IR and non-alcoholic fatty liver disease (NAFLD) in both obese pubertal children and adolescents [30,31]. Fat can accumulate also ectopically and improperly in different organs such as muscle, liver, pancreas, where fat-derived molecules foster a pro-inflammatory milieu [32].

### 2.1. Adipocyte-Fatty Acid-Binding Protein

A-FABP is a 132 amino acid cytoplasmatic lipid carrier produced by mature adipocytes, dendritic cells and macrophages. Its expression and secretion increases during adipogenesis [33,34]. Circulating levels of A-FABP were significantly higher in obese adult patients [35] with MetS [36]; and in NAFLD patients [37] when compared with healthy individuals. Studies in mice suggest that A-FABP promotes IR, increased levels of triacylglycerols (TAG), expression of pro-inflammatory genes and foam cell development, hence favoring atherosclerosis independently of weight gain [38]. Regarding the role of A-FABP in juvenile obesity, Corripio* et al.* [5] found significantly higher levels of the molecule in obese prepubertal children than in normal-weight children. A-FABP levels were correlated significantly with uric acid and ALT levels. In keeping with these findings, overweight Korean boys had significantly higher A-FABP concentrations than normal-weight boys. Moreover, baseline levels were significantly higher in those children who developed MetS 3 years later [6]. Among Korean children (9 years), the highest levels of A-FABP were detected in children with the worst metabolic phenotype,* i.e.*, children with greater body mass index (BMI), waist circumference (WC), higher levels of TAG, insulin, homeostasis model assessment of IR (HOMA-IR) and lower high density lipoprotein-cholesterol (HDL-c). Correlations of A-FABP with HOMA-IR and HDL-c remained significant even after adjusting for BMI [7]. In a sample of Polish children and adolescents, circulating levels of A-FABP were significantly higher in obese than in overweight patients and normal-weight age matched children. Moreover, in overweight and obese children, levels of A-FABP correlated positively with insulin, BMI, WC, TAG and HOMA-IR exclusively in girls, but with impaired 2-h glucose following the oral glucose tolerance test (OGTT) in both sexes. Levels of A-FABP correlated with HDL-c, high sensitivity C-reactive protein (hs-CRP) and leptin exclusively in boys [8]. In a small sample of obese Caucasian children at different pubertal stages, whose plasma levels of A-FABP were significantly higher than in normal-weight controls, Reinehr* et al.* [9] found no association with markers of MetS (*i.e.*, TAG, HDL-c and low density lipoprotein-cholesterol LDL-c, BP, HOMA-IR) and hs-CRP. Nevertheless, there was a positive correlation between levels of A-FABP and degrees of adiposity as estimated by the percentage of body fat and circulating leptin. On the contrary, Schipper* et al.* [10] observed no association between serum levels of A-FABP and obesity, although its levels were slightly increased in obese compared to lean children. It has been claimed that vitamin D deficiency/insufficiency, which are highly incidental among obese children, may contribute to low-grade inflammation and enhance cardiovascular risk, since the hormone exhibits immunomodulatory capacities. However, by comparing vitamin-D deficient (≤37.5 nmol/L) with (in)sufficient (insufficient 37.5–50 nmol/L; sufficient ≥ 50 nmol/L) obese and (in)sufficient obese with healthy children, no difference was found in levels of A-FABP [11]. Weight loss following a lifestyle intervention program determined a reduction of A-FABP levels. In obese children who lost weight (change of body mass index *Z* score ranging −0.7 to −0.5) after 1 year, A-FABP levels decreased significantly in parallel with the decrease of BMI Z score and leptin [9]. Krystek-Korpacka* et al.* [8] showed similar results in overweight/obese children and adolescents following a 1-year weight loss program. In a group of patients treated by metformin, levels of A-FABP were reduced, but not differently from that observed in the untreated group. Among prepubertal obese children who went through a 2-year weight loss program, those who lost considerable weight, showed a significant decrease of circulating A-FABP [5].

### 2.2. Chemerin

Chemerin is a 16-kDa chemattractant protein, ligand activator of the orphan G-protein coupled receptor chemokine-like receptor 1 [39]. It is mainly produced by the liver and WAT [40]. In humans, chemerin is significantly increased in obese and diabetic individuals; particularly in those with central adiposity, MetS [41], high levels of TAG, and reduced levels of adiponectin and HDL-c [42]. Chemerin modulates adipocytes expression of several pivotal genes involved in glucose and lipid metabolism (*i.e.*, glucose transporter-4, diacylglycerol *O*-acyltransferase-2) [40,43,44]. Cross-sectional studies in children have shown that serum levels of chemerin are significantly higher in obese children than those of normal-weight [10,12]. And particularly, in obese vitamin-D deficient children* vs.* not vitamin-D deficient and vitamin-D deficient obese patients, as previously defined in the A-FABP paragraph. The reason why chemerin levels are increased in obese vitamin-D deficient children remains unknown [11]. Chemerin levels were significantly correlated with BMI Z score, waist to hip ratio, skin fold thickness and serum leptin. Furthermore, values of chemerin, adjusted for age, sex and BMI, were significantly associated with some metabolic and cardiovascular abnormalities (systolic/diastolic BP, mean systolic 24 h BP, TAG, total cholesterol levels); with degrees of IR as estimated by levels of fasting insulin, HOMA-IR and insulin sensitivity index (ISI); with markers of inflammation such as hs-CRP, white blood cell count, and with markers of endothelium dysfunction such as intercellular adhesion molecule-1 and E-selectin [12], whose expression in human coronary artery endothelial cells is induced by chemerin [45].

### 2.3. Fibroblastic Growth Factor-21 

**Figure 2:**
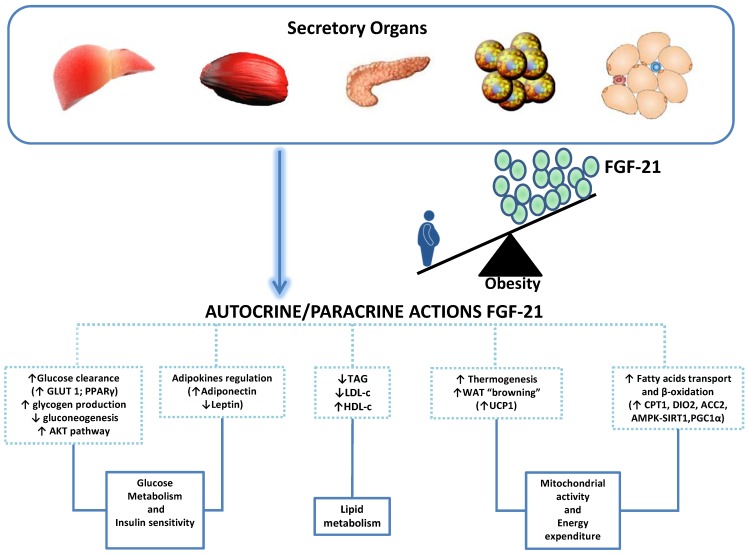
Metabolic functions of FGF-21. Abbreviations: glucose transporter-1 (GLUT-1); peroxisome proliferator-activated receptor gamma (PPARγ); protein kinase B (PKB or AKT); triacylglycerols (TAG); low density lipoprotein-cholesterol (LDL-c); high density lipoprotein-cholesterol (HDL-c); white adipose tissue (WAT); uncoupling protein 1 (UCP-1); carnitine palmitoyltransferase 1 (CPT1); type II iodothyronine deiodinase (DIO2); acetyl-CoA carboxylases 2 (ACC2); AMP-activated protein kinase—Sirtuin 1 (AMPK-SIRT1); peroxisome proliferator-activated receptor-γ coactivator-1 (PGC1α).

FGF-21 is a 181 amino acid peptide belonging to the human FGF family [46]. It is preferentially released by the liver and, to a minor extent, by skeletal muscle, thymus, pancreas, WAT and BAT [47,48]. As shown by* in vitro* and in animal studies, FGF21 stimulates lipolysis in WAT and ketogenesis in liver; it increases hepatic glycogen production and reduces gluconeogenesis [49]. FGF-21 significantly improves insulin signalling by enhancing phosphatidylinositol 3-kinase PI3K/AKT pathway, up-regulating glucose uptake and promoting the release of insulin-sensitizing adipokines such as adiponectin and reducing the release of insulin-antagonizing leptin [50]. FGF-21 is able to ameliorate dyslipidemia reducing TAG and LDL-c and increasing HDL-c [51]. It up-regulates WAT and BAT expression of uncoupling protein 1, a mitochondrial protein pivotal in the control of thermogenesis, enhancing the “browning” of WAT. It also up-regulates many mitochondrial genes, thus promoting beta-oxidation [52,53]. Therefore, FGF-21 has been proposed as a thermogenic; anti-hyperglycemic and anti-hyperlipidemic drug (Figure 2) [54]. Serum levels of FGF-21 were increased in obese adult patients [13,55] and children as compared to normal-weight age-matched children [14,56]. Interestingly, in children levels of FGF-21 showed a parallel increase with the grade of hepatic fat content independently of obesity development, visceral fat, and either hepatic or adipocyte IR [56]. Indeed, after adjusting for hepatic fat content, no association was found between serum concentrations of FGF-21 and hepatic and adipocytes IR indices (the product of endogenous glucose production per fasting insulin concentrations, and the product of plasma free fatty acids (FFAs) per fasting insulin concentration, respectively). Furthermore, serum levels of FGF-21 were positively correlated with circulating levels of citokeratine 18, a novel reliable marker of cellular apoptosis [57], and with the NAFLD activity score in 14 obese patients who underwent a liver biopsy [56]. On the contrary, Reinehr* et al.* [14] found no difference in FGF-21 levels in children with ultrasound evidence of fatty liver* vs.* children with no fatty liver as well as in those with or without MetS. However, Reinehr* et al.* [14] found a significant correlation between levels of FGF-21, degree of adiposity, circulating leptin and FFAs. Indeed, the i.v. infusion of FFAs (*i.e.*, oleic and linoleic acid) increased circulating levels of FGF-21 in healthy volunteers [58]. Circulating levels of FGF-21 decreased significantly following a 1-year lifestyle intervention program together with the reduction of BMI Z score, leptin and FFAs but independently of the amelioration of any parameter of the MetS, NAFLD and body composition [14].

### 2.4. Lipocalin-2

Lipocalin-2 is a 25 kDa protein belonging to the lipocalin family and is produced by immune cells mainly neutrophils, and adipocytes [59]. Circulating levels of lipocalin-2 were increased in adults with obesity and MetS [60,61]. Findings in obese children are still controversial. Studies reported weak correlations, often opposing and with uncertain clinical meaning. For instance, in a sample of 18 years-old overweight boys, Liu* et al.* [62] found a weak but positive association between circulating levels of lipocalin-2 and WC, TAG, and uric acid but not body weight and BMI. Adjusting for age, BMI, total body fat percentage, serum lipocalin-2 levels were significantly but inversely correlated with insulin and HOMA-IR. Then, the correlation disappeared after adjusting for creatinine levels. At 2-year follow-up, no correlation was found. In a small sample of obese and normal-weight children, a more recent pilot study did not reveal association with any anthropometric (BMI, BMI Z score) and cardio-metabolic parameter (HOMA-IR, TAG, total cholesterol, LDL-c, HDL-c) [15]. Kanaka-Gantembein* et al.* [16] found a significant negative correlation with BMI Z score values but no correlation with HOMA-IR in IR or T2DM obese girls. Serum lipocalin-2 was higher in prepubertal obese subjects in comparison with normal-weight ones, but circulating levels were not reduced following a 2-year weight loss program in obese cases [5].

### 2.5. Omentin-1

First described in the Paneth cells of the small intestine, omentin-1 is a 34 kDa protein, mainly produced by VAT, small intestine, heart, colon and thymus [63]. In primary human adipocytes, omentin-1 improves transduction of insulin signal, hence, enhancing insulin-stimulated glucose transport [64]. Indeed, plasma omentin-1 levels were significantly decreased in obese adults and, particularly, in patients with IR and T2DM, whose glucose transport is impaired [65,66]. Studies in childhood obesity showed inconsistent and, even, controversial data. Catli* et al.* [17] demonstrated that serum concentrations of omentin-1 were significantly lower in obese patients compared to normal-weight children and negatively correlated with BMI, WC, HOMA-IR and insulin levels. Prats-Puig* et al.* [67] found higher levels of omentin-1 in obese patients with more severe IR, higher TAG, systolic and diastolic BP, family history of diabetes and reduced levels of high-molecular weight adiponectin. On the contrary, Schipper* et al.* [10] did not find significant difference in levels of omentin-1 between normal weight and obese individuals, but just a positive trend in obese children. Moreover, in the study previously described (see Section 2.1), no difference in levels of omentin-1 was found among vitamin-D deficient obese, (in)sufficient obese and healthy children [11]. 

### 2.6. Vaspin

Vaspin stands for VAT derived serpin. It is a 395 amino acid protein belonging to the serine protease inhibitor family [68,69]. Evidence in obese and diabetic Otsuka Long-Evans Tokushima rats (OLEFT), suggests that vaspin improves insulin action and glucose tolerance. Its levels decreased significantly with the worsening of diabetes [69]. Data in humans is more controversial since some studies found VAT expression as well as circulating levels of vaspin significantly increased in obese adults. Both concentrations increased in parallel with the raising of BMI and the worsening of glucose tolerance [70,71,72]. A study found no difference in VAT expression and circulating levels of the molecule [59]. In 9-year overweight prepubertal Korean children [18] and in older obese children and adolescents [19], serum concentrations of vaspin were significantly higher than in normal-weight controls positively correlating with weight, BMI, diastolic BP [18], BMI Z score, TAG, insulin and HOMA-IR, and negatively with adiponectin and fasting glucose-to-insulin ratio [19]. Other studies by Korner* et al.* [20] and Martos Moreno* et al.* [21] did not confirm these findings. Indeed, Korner* et al.* [20] found serum levels of vaspin even lower in obese girls than in normal-weight girls without any correlation with BMI but with weak correlations with ISI and HOMA-IR after adjusting for sex, age and BMI Z score. Vaspin did not significantly increase with the pubertal phase in the obese subgroup. Serum concentrations of vaspin declined following OGTT in obese adolescents whose insulin peak during the load was higher than 1000 pmol/L [20]. Martos-Moreno* et al.* [21] observed a similar decrease following the OGTT despite that no difference was found between lean and obese pre-pubertal children in vaspin levels in both fasting and after load conditions. Vaspin levels decreased significantly in the short-term (7 days) following intensive lifestyle modification, being negatively correlated to insulin levels and HOMA-IR [73], but not in the long term following a standard weight loss program [21]. Vaspin might be involved in the pathogenesis and progression of cardiovascular disease since it is also expressed in periadventitial and epicardial adipose tissue as well as in vascular smooth cells [74]. Nevertheless, circulating vaspin was negatively correlated with 24-h systolic BP in obese children and reduced levels of vaspin were related to impaired endothelial function as estimated by the reactive hyperemia index [20]. 

## 3. Conclusions 

Early onset of obesity seems to shape a more severe pro-inflammatory phenotype than adult obesity, being associated with both hypertrophy and hyperplasia of the WAT. The massive recruitment of pre-adipocytes and their differentiation into mature adipocytes, particularly at the visceral site, causes the release of pro-inflammatory molecules able to act in a paracrine and systemical way on metabolically active organs. As for the recently discovered adipokines, evidence mainly based on cross-sectional studies suggests that A-FABP, chemerin and FGF-21 are significantly associated with early obesity and metabolic abnormalities. On the contrary, data on the role of lipocalin-2, vaspin and omentin-1 in the pathogenesis of childhood obesity is inconsistent. In particular, A-FABP seems to be associated with MetS; chemerin with IR and other cardiovascular abnormalities; FGF-21 with fatty liver. 

We strongly suggest that circulating levels of some adipokines may represent early biomarkers and/or risk factors to predict the persistence of obesity from childhood into adulthood. They could be useful to identify asymptomatic progression toward metabolic and cardiovascular abnormalities associated with obesity as well as to find new treatment options and to measure their efficacies. Nevertheless, taking into account that most of the studies are small and cross-sectional in design, and extremely heterogeneous in methodology used and population examined, longitudinal studies in large cohorts are needed to provide deeper insight into adipokines molecular mechanisms of action. Further, randomized clinical trials might be useful to better define the role of adipokines as biomarkers of therapeutic efficacy. 

## Abbreviations

A-FABP: adipocytes fatty acids binding protein
BAT: brown adipose tissue
BMI: body mass index
BP: blood pressure
FFA: free fatty acids
FGF-21: fibroblast growth factor-21
HDL-c: high density lipoprotein-cholesterol
HOMA-IR: homeostasis model assessment—insulin resistance
Hs-CRP: high sensitivity C-reactive protein
IR: insulin resistance
ISI: insulin sensitivity index
LDL-c: low density lipoprotein-cholesterol
MetS: metabolic syndrome
NAFLD: non-alcoholic fatty liver
OGTT: Oral glucose tolerance test
SAT: subcutaneous adipose tissue
T2DM: type II diabetes
TAG: triacylglycerols
VAT: visceral adipose tissue
WAT: white adipose tissue
WC: waist circumference
